# Testing personality in student selection: dispositional but not situational characteristics predict faking

**DOI:** 10.3389/fpsyg.2025.1592996

**Published:** 2025-05-19

**Authors:** Barbara Weissenbacher, Marcel Jud, Georg Krammer

**Affiliations:** ^1^Department of Differential Psychology, Institute of Psychology, University of Graz, Graz, Austria; ^2^Institute of Business and Vocational Education, Johannes Kepler University Linz, Linz, Austria

**Keywords:** faking, Big Five, student selection, pre-service teachers, dispositional characteristics, situational characteristics

## Abstract

The use of personality tests in selection procedures is controversial because of their susceptibility to faking. The purpose of this study was to examine the extent and prediction of faking, including dispositional and situational characteristics in a within-subjects design. We compared Big Five scores from a low-stakes and a high-stakes situation in candidates applying for initial teacher education. Across all Big Five traits, participants scored significantly higher in the high-stakes situation than in the low-stakes situation. We found that the extent of faking depended on the personality trait, with high effect sizes in emotional stability (*d* = 0.94) and low effect sizes in extraversion (*d* = 0.29) and agreeableness (*d* = 0.19). Results from hierarchical regression analyses indicated that male gender and intelligence positively predicted faking in certain personality traits. Unexpectedly, emotional stability and conscientiousness also positively predicted faking. Situational factors could not explain incremental variance in the criteria over and above the dispositional factors. Overall, the amount of variance explained in faking was low, stressing that we still know too little about the interindividual differences in faking and thus to address faking as a systematic process.

## 1 Introduction

The use of personality tests in selection procedures has been a subject of considerable debate due to the tension between high validity and high susceptibility to faking (Rothstein and Goffin, 2006; Morgeson et al., 2007). Personality traits show predictive validity for academic and job success over and above cognitive abilities (e.g., Barrick et al., 2001; Poropat, 2009; Mammadov, 2022). Meta-analyses on academic achievement emphasize the impact of conscientiousness, which contributes significantly across different levels of education, independently of the measure used (Poropat, 2009; McAbee and Oswald, 2013). Even for job success, conscientiousness shows predictive validity, across performance measures and occupations; beyond the global effect of conscientiousness, the predictive validity of the other Big Five traits varies across occupations (Barrick et al., 2001).

Even though personality traits exhibit predictive validity for academic and job success and could therefore be consistently used in student or personnel selection, they have a major disadvantage: personality tests mostly rely on self-report measures which are prone to faking in high-stakes-situations. To enhance their selection chances, applicants may present themselves in an overly favorable light, choosing responses they believe are desirable rather than responses that accurately reflect their personality (e.g., Birkeland et al., 2006). Hu and Connelly (2021) meta-analyzed 20 within-subject design studies that included personality measures both in a high-stakes and a low-stakes situation. They found that applicants had moderately higher means, slightly reduced variability, and stronger rank-order consistency in the high-stakes (application) situation compared to the low-stakes situation. This tendency to fake can undermine the reliability and validity of the test results (Salgado, 2018; Krammer et al., 2025; Speer et al., 2025) and therefore the correctness of the selection decision.

In the prediction of faking behavior both dispositional (individual) and situational (contextual) variables play a role (Snell et al., 1999), though the extent of each impact varies across different studies and contexts. Levashina and Campion (2006) developed a model of faking in employment interviews that describes faking as a function of capacity, willingness, and opportunity to fake; all three elements must be met for faking to occur. *Capacity* includes, among others, social skills, cognitive abilities, and knowledge of the construct being measured. *Willingness to fake* consists of individual traits like extraversion, agreeableness or integrity. *Opportunity to fake* refers to the interview structure and constructs being assessed.

Regarding dispositional characteristics, cognitive abilities – which, according to Levashina and Campion (2006), belong to *capacity* – have emerged to correlate positively with faking behavior (Pauls and Crost, 2005; Tett et al., 2012; Dunlop et al., 2022). Schilling et al. (2021) meta-analytically showed that personality scores correlated higher with cognitive abilities when assessed in selection situations compared to non-selective situations, which can be seen as indirect evidence of faking affecting personality assessment. A certain level of intelligence is necessary to “fake successfully” (Geiger et al., 2018), this is also called “ability to fake.” The ability to fake reflects a main component of McFarland and Ryan’s model of faking (2000). Pauls and Crost (2005) found that more intelligent individuals are better in perceiving the situational requirements and recognizing the meaning of a certain personality item, which leads to a higher ability to fake, and therefore to higher faking.

Personality traits – which are associated with the *willingness to fake* – also contribute to the prediction of faking. McFarland and Ryan (2000) examined undergraduate students in a within-subjects design and found that – among the Big Five – neuroticism and conscientiousness significantly predicted faking. Individuals scoring higher on neuroticism and lower on conscientiousness faked to a greater extent. This is explained by the fact that conscientious individuals tend to observe the rules and behave responsibly. Individuals scoring high on neuroticism are concerned about how others see them which might lead to higher impression management. The importance of conscientiousness in faking could also be shown in a study of Bill et al. (2020) in which they examined situational and dispositional antecedents of faking intentions in selection interviews. Of the Big Five, only conscientiousness was moderately correlated with faking intention; the other Big Five factors did not contribute to the prediction. Among the other Big Five factors, most studies did not show relationships with faking (e.g., McFarland and Ryan, 2000; Bill et al., 2020).

Gender and age also play a role in the *willingness to fake*, although there is little research on the relationship with faking. As a study by Hogue et al. (2013) has shown, men tend to fake more than women which is explained by women’s higher ethical standards and men’s greater lying behavior to secure monetary benefits (Volkema, 2004; Dreber and Johannesson, 2008). On the other hand, it has been shown that social desirability plays a greater role for women; for example, they score higher in impression management (Bernardi and Guptill, 2008). A recent review of selection interview faking studies by Melchers et al. (2020) suggested that age was negatively associated with faking frequency.

Looking at situational characteristics (influencing the *opportunity to fake*), studies have shown that the relevance attributed to specific traits required for the present profession moderates the extent to which individuals engage in faking. Tett et al. (2012) showed in a sample of undergraduates that faking was 26 percent greater on job-relevant traits (independently rated by subject matter experts). Krammer (2020) examined applicants for teacher education and yielded the result that items’ perceived relevance for professional purposes was related to faking.

Ellingson and McFarland (2011) proposed a framework for understanding how perceptions of desirability, perceived outcomes, and expectations of success influence faking in high-stakes situations, the VIE (valence-instrumentality-expectancy) theory. The theory suggests that applicants are more likely to engage in faking behavior when they value the outcome highly (valence), believe that their faking will directly contribute to achieving that outcome (instrumentality), and feel capable of faking effectively (expectancy). Dunlop et al. (2022) found that faking in an experimental high-stakes situation could be predicted best by perceived job desirability (valence), followed by expectancy and instrumentality. Examining the three predictors together in a linear regression model, only valence significantly contributed to the prediction of faking.

While many previous studies have examined either specific dispositional or situational characteristics in relation to faking, we aim to investigate multiple dispositional and situational factors together to help develop a deeper understanding of the antecedents of faking. By identifying the factors that contribute to faking behavior in selection procedures, learning more about which people fake more or less in which situations can help make selection procedures fairer and improve the reliability and validity of personality tests used.

In this study, we examine the extent and prediction of faking, including dispositional and situational characteristics in a within-subjects design. By comparing Big Five scores between low-stakes and high-stakes situations for candidates applying to initial teacher education, we aim to address the following research questions (RQ) and hypotheses (H) based on the literature reviewed above:

(1) Do people in the high-stakes situation exhibit higher scores in the Big Five than in the low-stakes situation and how large are the effects?

*H1*: Mean scores on all personality domains are higher in the high-stakes situation than in the low-stakes situation.

(2) How can dispositional characteristics contribute to the prediction of faking?

(RQ1) Do age and gender predict faking?

*H2*: Conscientiousness and emotional stability negatively predict faking.

*H3*: Intelligence positively predicts faking.

(3) How can situational characteristics contribute to the prediction of faking?

*H4*: Perceived relevance of a trait positively predicts faking in that trait.

*H5*: Valence positively predicts faking.

(RQ2a) Does instrumentality positively predict faking?

(RQ2b) Does expectancy positively predict faking?

## 2 Materials and method

### 2.1 Participants

At time 1 (t1), 561 people participated (representing about a quarter of those who took the admission exam). Because not all of them eventually took the admission exam, some codes were incorrect, and some data were incomplete, the final sample (i.e., merged data from both measurement points) included 448 people. Of the participants, 77.46% identified themselves as female, 22.55% as male, and no one identified as diverse. The age ranged between 17 and 54 (*M* = 22.46, *SD* = 7.17). During the registration process, applicants decided if their admission exam data (time 2; t2) could be used for (future) scientific purposes. If they did not agree (which applied to 9.53% of the applicants), they could still participate in the admission exam, but their data were not used in the present study. In the voluntary online study (t1), participants were informed and had to approve that their data could be used (anonymously and not on an individual basis) in scientific publications and might also be published in suitable online repositories. Participants were not rewarded.

### 2.2 Design and procedure

In our study, faking was examined in a within-subject design. Prior to the admission exam for initial teacher education, we asked the applicants (during the registration process) to participate in a voluntary online study (t1) which is not part of the admission procedure (and does not affect the results of the admission exam). T1 represented a low-stakes situation; we expected participants to answer rather honestly. T2 consisted of the admission exam (as part of the selection procedure for pre-service teachers) and represented a high-stakes situation where we expected participants to fake-good. Participation at t2 was obligatory since all candidates applying for initial teacher education have to complete the multi-stage admission procedure TESAT (Teacher Student Assessment Austria; Neubauer et al., 2017). The first stage included a non-selective (but obligatory) online self-assessment, the second stage a computer-based admission exam. In this study, data from the computer-based admission exam were used.

The data of t1 have been merged with the data of t2 by the individual code the participants were asked to generate. Since there were two time slots for the registration for teacher education studies (March to May and July to August) and the admission exam (June to July and August), data collection started in March and ended in August 2024.

### 2.3 Instruments

#### 2.3.1 Personality

At t1, personality was assessed using a short version of the German Big Five Inventory (BFI-K) by Rammstedt and John (2005). It included the dimensions openness, extraversion, agreeableness, conscientiousness, and neuroticism with 4–5 items each on a five-point scale. The wording of some items was adapted to that of the 42-item version of the German Big Five Inventory (BFI; Lang et al., 2001) which was used at t2. Even though the Big Five were assessed in more detail in t2, due to comparability, we only used the same 4–5 items as in t1 to calculate the mean scores in this study. At t1, the internal consistencies ranged from α = 0.60 to α = 0.76. At t2, the internal consistencies ranged from α = 0.62 to α = 0.77.

#### 2.3.2 Cognitive abilities

To assess cognitive abilities at t2, self-developed scales for verbal, numeric and figural abilities were used. Verbal abilities were measured using 21 similarity items. In each item, five words were presented; the participants had to decide which word did not fit the others. For numeric abilities, we asked participants to identify the rules on which a number sequence is based and use them to continue the sequence; they had to answer 14 of these items. For measuring figural abilities, the participants completed 16 paper folding items where diagrams – of a paper being folded and a hole being punched – were presented. The participants had to identify the resulting punch pattern out of 5 answer options. For each scale, the number of correct items was summed up and a z-standardized mean score across the three scales was computed. The internal consistencies ranged between α = 0.79 (figural) and α = 0.86 (numeric). The instrument indicates satisfactory construct validity, as indicated by high correlations (0.49 ≤ *r* ≤ 0.52) with the corresponding scales of the Intelligence Structure Analysis (Blum et al., 1998). A moderate relationship between the mean score and previous school achievement (*r* = 0.41) supports the assumption of criterion validity.

#### 2.3.3 Valence, instrumentality and expectancy

To assess valence, instrumentality and expectancy (VIE), we adapted items from the test-taking expectancy motivation subscale (Sanchez et al., 2000) and translated them into German. 3 items were used for valence, 2 items were used for instrumentality, and 2 items for expectancy, each on a five-point scale. The internal consistencies were α = 0.58 (valence), α = 0.68 (instrumentality), and α = 0.68 (expectancy). The items are available on the project’s OSF page at https://osf.io/b5t8z/.

#### 2.3.4 Perceived relevance

We asked the participants to rate the perceived relevance of the Big Five traits (“How important do you consider the following characteristics to be for pre-service and in-service teachers?”) on a five-point scale (1 = not relevant, 5 = relevant). The Big Five were each described in more detail using three adjectives (e.g., for conscientiousness, “reliable, hard-working, organized”).

### 2.4 Statistical analyses

All statistical analyses were performed in R using the packages psych (Revelle, 2005), stats (R Core Team, 2022) and lsr (Navarro, 2015). For the analysis of mean shifts from t1 to t2, we looked at raw difference scores. To examine the prediction of faking, we used regression-adjusted difference scores (RADS; as suggested in Burns and Christiansen, 2011). In doing so, we regressed the high-stakes scores (t2) on the low-stakes scores (t1) for all Big Five traits and stored the standardized residuals. The R script is available on the project’s OSF page at https://osf.io/b5t8z/.

## 3 Results

### 3.1 Extent of faking

First, we analyzed the mean shift in the Big Five scores between t1 and t2 (*H1*). As expected, participants scored significantly higher in the high-stakes situation than in the low-stakes situation across all traits. The highest effect size was observed in emotional stability (*d* = 0.94). The other traits showed either low effect sizes (extraversion and agreeableness) or moderate effect sizes (openness and conscientiousness). Descriptive statistics as well as effect sizes and results of t-tests are presented in Table 1. Intercorrelations between all variables can be found in Supplementary Table 1.

**Table 1:** Comparison of Big Five scores between t1 and t2.

	*M*	*SD*	*Min*	*Max*	**α**	t-test	*d*
t1 Openness	4.23	0.53	2.80	5.00	0.62	*t*_447_ = −6.94, 95% CI [−0.17, −0.10]	0.33
t2 Openness	4.37	0.47	2.80	5.00	0.64		
t1 Conscientiousness	4.30	0.52	2.75	5.00	0.70	*t*_447_ = −6.33, 95% CI [−0.18, −0.09]	0.30
t2 Conscientiousness	4.44	0.46	3.00	5.00	0.70		
t1 Extraversion	4.06	0.65	1.50	5.00	0.77	*t*_447_ = −6.17, 95% CI [−0.19, −0.10]	0.29
t2 Extraversion	4.20	0.57	2.25	5.00	0.76		
t1 Agreeableness	4.07	0.59	1.75	5.00	0.62	*t*_447_ = −4.01, 95% CI [−0.15, −0.05]	0.19
t2 Agreeableness	4.18	0.54	2.00	5.00	0.60		
t1 Emotional Stability	3.48	0.67	1.00	4.75	0.71	*t*_447_ = −19.47, 95% CI [−0.53, −0.44]	0.92
t2 Emotional Stability	3.97	0.60	1.75	5.00	0.72		

*N* = 448. Paired t-test. t1 = low-stakes situation, t2 = high-stakes situation.

### 3.2 Prediction of faking

Next, we conducted linear hierarchical regression analyses to predict the faking of each Big Five factor (operationalized by RADS) from dispositional and situational variables. In each model, the dispositional factors (gender, age, conscientiousness (t1), emotional stability (t1), and intelligence) were included in the first step. In the prediction of the faking score for conscientiousness, we did not include conscientiousness (t1) to prevent circularity. For emotional stability, we proceeded in the same way. The situational factors (valence, instrumentality, expectancy and perceived relevance) were included in the second step.

The results (see Table 2) indicated rather low amounts of explained variances for all factors. Emotional stability could be predicted best (*R^2^* = 0.067), whereas the amount of explained variance in extraversion and agreeableness did not even exceed the significance threshold. The results of the hierarchical regression analyses indicated that situational factors could not explain incremental variance in the criteria over and above the dispositional factors.

**Table 2:** Hierarchical regression analyses predicting faking on the Big Five.

		Openness		Conscientiousness		Extraversion		Agreeableness		Emotional stability	
		*β*	95% CI	*β*	95% CI	*β*	95% CI	*β*	95% CI	*β*	95% CI
Step 1 (dispositional variables)	Gender	0.02	[−0.07, 0.12]	−0.06	[−0.16, 0.03]	0.06	[−0.04, 0.15]	−0.10	[−0.19, 0.00]	**0.13**	**[0.04, 0.22]**
	Age	−0.03	[−0.12, 0.07]	−0.04	[−0.13, 0.06]	**−0.11**	**[−0.21, −0.02]**	−0.02	[−0.12, 0.07]	0.04	[−0.05, 0.13]
	C	−0.05	[−0.15, 0.05]			0.10	[−0.00, 0.20]	0.07	[−0.03, 0.17]	**0.13**	**[0.04, 0.22]**
	ES	−0.05	[−0.15, 0.05]	**0.13**	**[0.04, 0.23]**	0.04	[−0.06, 0.14]	**0.10**	**[0.00, 0.20]**		
	IQ	0.09	[−0.01, 0.19]	**0.14**	**[0.05, 0.24]**	0.06	[−0.04, 0.15]	0.04	[−0.05, 0.14]	**0.15**	**[0.05, 0.24]**
	Model Fit	*R^2^* = 0.019 95% CI [0.00, 0.04]		*R^2^* = 0.033 95% CI [0.00, 0.06]		*R^2^* = 0.028 95% CI [0.00, 0.05]		*R^2^* = 0.025 95% CI [0.00, 0.05]		*R^2^* = 0.060 95% CI [0.02, 0.10]	
Step 2 (dispositional + situational variables)	Gender	0.04	[−0.06, 0.13]	−0.05	[−0.15, 0.05]	0.06	[−0.04, 0.16]	−0.09	[−0.19, 0.01]	**0.13**	**[0.04, 0.23]**
	Age	−0.02	[−0.12, 0.07]	−0.03	[−0.13, 0.07]	**−0.10**	**[−0.20, −0.00]**	−0.01	[−0.10, 0.09]	0.02	[−0.07, 0.12]
	C	−0.06	[−0.16, 0.04]			0.09	[−0.01, 0.19]	0.05	[−0.05, 0.15]	**0.13**	**[0.03, 0.22]**
	ES	−0.06	[−0.16, 0.04]	**0.12**	**[0.03, 0.22]**	0.04	[−0.06, 0.14]	0.09	[−0.01, 0.20]		
	IQ	0.10	[−0.00, 0.19]	**0.14**	**[0.05, 0.24]**	0.06	[−0.04, 0.16]	0.04	[−0.05, 0.14]	**0.14**	**[0.04, 0.23]**
	Valence	−0.04	[−0.14, 0.06]	0.03	[−0.06, 0.13]	0.05	[−0.05, 0.15]	**0.11**	**[0.01, 0.21]**	0.02	[−0.08, 0.11]
	Instrument	0.01	[−0.09, 0.12]	0.03	[−0.07, 0.14]	−0.03	[−0.13, 0.08]	0.09	[−0.02, 0.19]	−0.01	[−0.11, 0.10]
	Expectancy	0.03	[−0.08, 0.14]	−0.02	[−0.13, 0.09]	0.01	[−0.10, 0.12]	−0.03	[−0.14, 0.07]	−0.09	[−0.19, 0.02]
	Relevance	0.08	[−0.02, 0.18]	0.03	[−0.07, 0.12]	0.02	[−0.08, 0.12]	−0.02	[−0.11, 0.08]	−0.00	[−0.09, 0.09]
	Model Fit	*R^2^* = 0.027 95% CI [0.00, 0.04]		*R^2^* = 0.035 95% CI [0.00, 0.06]		*R^2^* = 0.031 95% CI [0.00, 0.05]		*R^2^* = 0.041 95% CI [0.00, 0.06]		*R^2^* = 0.067 95% CI [0.02, 0.10]	
	Difference	Δ*R^2^* = 0.008 95% CI [−0.01, 0.02]		Δ*R^2^* = 0.003 95% CI [−0.01, 0.01]		Δ*R^2^* = 0.003 95% CI [−0.01, 0.01]		Δ*R^2^* = 0.017 95% CI [−0.01, 0.04]		Δ*R^2^* = 0.007 95% CI [−0.01, 0.02]	

Bold print indicates statistical significance. Criteria (dependent variables) represent regression-adjusted difference scores. C=Conscientiousness, ES = Emotional stability.

Looking at dispositional predictors (Table 2), gender predicted faking on emotional stability in the way that males showed higher faking (*RQ1*). Age negatively predicted faking on extraversion (*RQ1*). Contrary to our hypotheses, conscientiousness positively predicted faking on emotional stability (*H2*); and emotional stability, in turn, also positively predicted faking on conscientiousness and agreeableness (*H3*). Intelligence predicted faking on conscientiousness and emotional stability (*H4*). Among the situational predictors, only valence was associated with faking; it significantly predicted agreeableness (*H5*), whereas instrumentality (RQ2a), expectancy (RQ2b) and perceived relevance (*H4*) did not contribute to the prediction.

## 4 Discussion

To examine the extent of faking and its prediction on personality tests in student selection, we compared results from a low-stakes situation with results from a high-stakes situation – the results of an admission exam. Our results provide evidence that the extent of faking depends on the personality trait, with high faking on emotional stability and low faking on extraversion and agreeableness. Results from hierarchical regressions indicated that dispositional factors explained some variance in faking, but situational factors could not explain incremental variance in the criteria over and above dispositional factors.

In line with previous studies (Birkeland et al., 2006; Hu and Connelly, 2021), participants scored significantly higher in the high-stakes situation than in the low-stakes situation across all Big Five traits with the highest effect size in emotional stability. The small effect sizes in extraversion and agreeableness may reflect less malleability in these traits under impression management. It may also be the case that applicants assume that scoring too high on extraversion and agreeableness is not desirable: for example, given the requirements of studying at a university, it may be considered desirable not to be too assertive and outgoing. In contrast, the high susceptibility of emotional stability to faking suggests that applicants may perceive this trait as especially valued by employers and, as a result, may concentrate on enhancing their scores (Birkeland et al., 2006). The effect sizes were comparable to those of previous within-subject studies with the order low-stakes situation before high-stakes situation (Hu and Connelly, 2021). While faking can be seen as an undesirable deception from an organizational (in this case a university) perspective because it prevents us from getting the “true” personality scores of a person, it can also be seen from a self-presentation perspective: as Marcus (2009) argues, from the applicant’s perspective, adapting one’s responses in a high-stakes situation can be seen as an adaptation to situational demands which is rather positive.

The hierarchical regression analyses revealed that dispositional factors explained some variance in faking, though situational factors added little incremental predictive power. Emotional stability emerged not only as the trait most susceptible to faking, but also as the best-predicted trait, with intelligence, gender and conscientiousness contributing to faking behavior. In terms of demographic characteristics, gender and age showed small effects. Men faked more on emotional stability, and older people faked less on extraversion. Our results are consistent with previous studies (Hogue et al., 2013; Melchers et al., 2020), but the effects found are negligible so we can conclude that gender and age appear to have little effect on the extent of faking.

Contrary to our hypotheses, conscientiousness and emotional stability positively, rather than negatively, predicted faking on certain traits (albeit to a small extent). Emotional stability predicted faking on conscientiousness and agreeableness, while conscientiousness predicted faking on emotional stability. These unexpected findings suggest that individuals higher in these traits might possess greater social insight or strategic awareness, enabling them to tailor their responses effectively. Even if this result contradicts previous studies (McFarland and Ryan, 2000; Bill et al., 2020), it can still be explained. As Goffin and Boyd (2009) argue, only certain facets of conscientiousness and emotional stability are associated with a lower motivation to fake (which precedes faking), while others increase faking. For example, dutifulness is expected to correlate negatively with the motivation to fake because dutiful people are more likely to follow instructions. In contrast, achievement striving is expected to correlate positively with the motivation to fake (due to the high ambition to get a certain job; or in our case: to pass the admission exam). Among the facets of emotional stability, low anxiety (or in other words, calmness) may lead to lower motivation to fake because people see no need to fake due to their optimistic attitudes; the facet of impulsiveness, on the other hand, may be more positively associated with motivation to fake because of reduced control over the urge to fake a response to an item.

At least for the traits conscientiousness and emotional stability, intelligence was – as expected – a significant predictor of faking, albeit a weak one. This result, which is align with previous studies (Pauls and Crost, 2005; Dunlop et al., 2022), indicates that more intelligent people tend to fake more (or fake more successfully).

The hypothesis that perceived relevance would positively predict faking was not supported. While a previous study by Krammer (2020) has found correlations between the perceived relevance and the extent of faking in that trait, this was not the case for any of the Big Five traits in our study. An explanation for this hypothesis-defying result could lie in our study design: relevance was asked at the trait level and not – as in Krammer (2020)'s study – at the item level. It is possible that participants in our study found it more difficult to establish the connection between the trait perceived as desirable and the corresponding items.

Among the VIE predictors, only valence significantly predicted faking (but only in agreeableness). Valence being the only predictive factor is consistent with the findings of Dunlop et al. (2022) who found that only valence contributed to the prediction, although instrumentality and expectancy showed zero-order correlations with faking. One explanation for why valence was not as predictive as expected might be the low variance: the vast majority of people reported that passing the admission exam was very important to them (*M* = 4.78, *SD* = 0.45).

### 4.1 Limitations

Although we included a large number of dispositional and situational traits in this study, we were still unable to explain much of the variance in faking. Several factors could be responsible for this: (1) In the sample on which our study on actual applicants for initial teacher education is based, the average scores of the Big Five generally deviated from those of a typical population; this applies not only to the high-stakes situation, but also to the low-stakes situation. Comparing the scores of our low-stakes situation to the scores of a student sample reported by Rammstedt and John (2005), our participants scored about half a standard deviation higher on all traits; and in the high-stakes situation, ceiling effects occurred. However, the surprisingly high means can be partly explained by the study program. As Hartmann and Ertl (2023) have shown, pre-service teachers score – compared to students from other study programs – significantly higher on extraversion and, in some cases (depending on gender and subject), also on emotional stability and agreeableness. This suggests that the means found in our study are quite comparable within the group of (future) pre-service teachers. (2) Researchers in previous faking studies have already pointed out (e.g., Holtrop et al., 2021; Krammer et al., 2025) that we do not know whether the answers given in the condition in which we expect them to be honest are really honest – or whether, for example, applicants also give socially desirable answers in the low-stakes situation. In our study, it would be possible that participants were skeptical that the answers they give in the low-stakes situation would be included in the admission exam score (although we made it clear that this was not the case). If they were to fake their answers at t1 as well, this would limit the interpretability of all non-cognitive predictors. (3) Regarding situational factors, the assessment was not ideal. To investigate the relationship between perceived relevance and faking, it would be more useful to ask about relevance at the item level and separately for study relevance and job relevance (cf. Krammer, 2020).

Another limitation arises from the design of the study: because we could not randomize t1 and t2 in our sample of real applicants (t1 was always the low-stakes situation and t2 always the high-stakes situation), carry-over and practice effects may have occurred. It may be that the answers given at t2 are influenced by the fact that some of the items were already presented at t1. To minimize these effects, we did not present the same questionnaire twice but used a shortened version of the BFI (Lang et al., 2001) at t1.

### 4.2 Implications and conclusions

Our results confirm the findings of numerous previous studies that faking occurs in high-stakes situations, particularly for the trait emotional stability (e.g., Birkeland et al., 2006; Hu and Connelly, 2021). This suggests that, in selection contexts, self-reported scores of emotional stability may not fully reflect a candidate’s true dispositional profile. In practice, (high) emotional stability scores may be interpreted more cautiously – especially when they are not accompanied by converging evidence from other assessment methods (e.g., interviews). To adjust the evaluative focus, admission committees might, for example, consider placing relatively less weight on self-reported emotional stability and more on traits that show robust predictive validity, such as conscientiousness, which appears less prone to faking.

Dispositional factors seem to be primarily responsible for faking rather than situational factors in the context of university admission exams. However, the variance explained by these factors is small. As in previous studies (e.g., Krammer, 2020), this study has again shown that – at least within selection procedures – faking does not appear to be a systematic process, but rather a construct of interindividual differences, which in turn cannot be explained simply by interindividual differences. In practice, our finding that traits such as gender, conscientiousness, emotional stability, and intelligence are poor predictors of faking is not at all negative. Much more problematic would be the result that less conscientious, highly neurotic, highly intelligent, and male individuals would systematically fake their personality test results and thus gain an unfair advantage in the admission exam.

## Data Availability

The raw data supporting the conclusions of this article will be made available by the authors, without undue reservation.
